# Gene variants polymorphisms and uterine leiomyoma: an updated review

**DOI:** 10.3389/fgene.2024.1330807

**Published:** 2024-03-20

**Authors:** Sonal Upadhyay, Pawan K. Dubey

**Affiliations:** Centre for Genetic Disorders, Institute of Science, Banaras Hindu University, Varanasi, Uttar Pradesh, India

**Keywords:** Uterine leiomyoma, single nucleotide polymorphism, biomarker, genetic variants, fibroid

## Abstract

Uterine leiomyoma, commonly referred to as fibroids, is a benign tumor that develops in the muscular wall of the uterus. These growths are non-cancerous and can vary in size, ranging from tiny nodules to larger masses. Uterine leiomyomas often occur during a woman’s reproductive years and can lead to symptoms such as heavy menstrual bleeding, pelvic pain, and pressure on nearby organs. While the exact cause is not fully understood, hormonal factors, particularly estrogen and progesterone, are believed to play a role in their development. The exploration of connections between genetic variants and uterine leiomyoma has captivated scientific attention for numerous years. The results from investigations remain a subject of intrigue within the scientific community. To date, the findings regarding the relationships between single nucleotide polymorphisms (SNPs) and uterine leiomyoma have exhibited some inconsistencies. However, amidst these inconsistencies, several promising outcomes have emerged that hold the potential to shape future research endeavors. These promising leads could pave the way for the development of innovative targeted therapies and novel prognostic biomarkers. This review specifically centers on accentuating the existing literature data concerning genetic variants that have been explored for their potential connections to uterine leiomyoma. Additionally, it underscores the prospects of employing genetic variations as diagnostic and prognostic biomarkers for individuals diagnosed with uterine leiomyoma.

## Introduction

Originally referred to as “uterine stones,” uterine leiomyoma (UL) lesions were later known as “scleromas” during the second century AD. The term “leiomyoma” was introduced in the 1860s. Today, uterine leiomyoma also known as uterine fibroid are recognized as the most prevalent pelvic tumors among women of reproductive age, impacting over 70% of women worldwide, with a higher incidence among women of color. These leiomyoma exhibit a diverse range of composition, size, and quantity both among women and within the same individual (Baird et al., 2003; Stewart et al., 2016; Wise and Laughlin-Tommaso, 2016).

The occurrence of uterine fibroids is on the rise in specific populations, notably among African American women. Race is the primary and commonly cited risk factor, affecting African American women at a higher rate compared to other groups. Additionally, other risk factors for uterine fibroids include advancing age, being in a premenopausal state, not having given birth, having a family history of uterine fibroids, hypertension, consumption of food additives, and frequent intake of soybean milk (Al-Hendy et al., 2017).

Unlike genetic mutations, which refer to abnormal nucleotide changes in a DNA sequence, that is frequently observed within the population which might have some clinical significance in genesis of uterine leiomyoma. In recent times, molecular biomarkers such as Somatic mutations in the Xq13 gene that encodes the RNA Polymerase II (Pol II) mediator subunit MED12 are the most common, accounting for 45%–90% of uterine leiomyoma cases, with prevalence varying based on patient ethnicity (Mäkinen et al., 2011; Mehine et al., 2014). Further, it is also reported that genetic alterations that result in HMGA2 over-expression, disruption of the COL4A5-COL4A6 locus, and biallelic loss of FH, which encodes the tricarboxylic acid (TCA) cycle enzyme fumarate hydratase are associated with uterine leiomyoma (Tomlinson et al., 2002; Kämpjärvi et al., 2014; Pandey et al., 2024).

Although the causes and mechanisms of uterine leiomyoma’s (UL) etiology remain mostly unknown, but several studies have reported multi-factorial origin including genetic, hormonal, and environmental factors that contributes in genesis of uterine leiomyoma. The studies related to chromosomal and molecular analyses have provided data that support the role of genetic factors in the pathogenesis of uterine leiomyoma (Baird et al., 2015). Moreover, environmental and lifestyle variables, including nutrition, exposure to high endocrine disruptors (EDCs), vitamin D insufficiency, caffeine and alcohol intake, smoking, physical exercise, and stress, have also contributed in genesis of ULs (Bulun, 2013; Islam et al., 2013). Further, several other pathogenic factors such as genetics, epigenetics, steroid sex hormones, growth factors, cytokines, and chemokines, excessive deposition of extracellular matrix (ECM) components, and matrix metalloproteinases (MMPs) are implicated in UF remodeling than in surrounding myometrium have a role in the UF development and growth (Markowski et al., 2012; Segars et al., 2014; Maltese et al., 2018).

Cytogenetic studies have shown that approximately 40%–50% of UF harbor chromosomal abnormalities and linked to changes in a number of genes, including MED12, HMGA2, COL4A5/COL4A6, and FH genes (McGuire et al., 2012; Segars et al., 2014). Studies have demonstrated that from total genetic aberration, about 70%–80% of uterine leiomyoma’s shown site-specific somatic mutation on mediator complex subunit 12 (*MED12*) genes in different populations, including Finnish (Caucasian), northern United States regions, and South African women (Mittal et al., 2015; Noutakdie Tochie et al., 2021). These studies suggest the need to have ethnic or population specific genetic data to gain insight into the disease etiology. *MED12* gene belongs to the mediator complex, which consists of 26 subunit transcriptional regulators that bridge the DNA regulatory sequences to modulates RNA polymerase II activity, and involved in global gene transcriptional regulation within cells. It is reported that heterozygous missense and frame-shift mutation on exon-1 and exon-2 of *MED12* have the most frequent genetic instability in UL pathogenesis (Cardozo et al., 2012; Baird et al., 2015). Somatic mutations in exon-2 of the *MED12* gene have been recently reported by several studies However, the prevalence of *MED12* gene mutations and the functional roles of these mutations in the tumorigenesis of uterine fibroids in north Indian population remain unknown.

Estimates suggest that uterine leiomyoma incur approximately $34 billion in annual healthcare costs in the United States alone. As a result, these leiomyoma impose a considerable burden on both societal health and financial aspects. The primary objective of this narrative review is to underscore the current literature on genetic variants explored for their potential link with uterine leiomyoma. Additionally, it aims to emphasize the potential of utilizing genetic variations as biomarkers for diagnosis and prognosis of uterine leiomyoma patients.

## Methodology

### Data sources and search strategy

The PRISMA (Preferred Reporting Items for Systematic Reviews and Meta-Analyses) framework was used to perform this review. A widespread systematic literature review with dates between 2001 and 2023 using keywords with “Uterine fibroid, Uterine leiomyoma Single nucleotide polymorphism, genetic variant, and Genetic variants in databases like PubMed, Scopus, Web of Science, and Google Scholar were carried out by authors. Furthermore, the reference lists of significant publications were examined to identify additional relevant articles. All the reviewed papers were in English, and data pertinent to the topic of this article were extracted.

### Single nucleotide variant and Uterine leiomyoma

Genetic analysis results can significantly influence the advancement, prognosis, and management of various diseases, with cancer being particularly noteworthy due to the vast array of genetic alterations commonly linked to it.

Among genetic variations, the most prevalent type is single nucleotide polymorphism (SNP). SNPs involve variations in the DNA sequence at a single nucleotide level and can result from point mutations, deletions, insertions, or base-pair repeats. Detecting SNPs is a common and straightforward method for identifying genetic variations, making them a key focus in genetic research. SNPs are typically classified as “common” if their frequency is ≥ 1% in the general population, while those below this value are considered “rare.” Some authors consider SNPs as “common” with a frequency ≥5%, “rare” between 1% and 5%, and “subpolymorphic” with a frequency ≤1% (Carvajal-Carmona, 2010; Saenko and Rogounovitch, 2018; Huang et al., 2021). Many SNPs are considered benign as they do not significantly impact corresponding genes, contributing to human population diversity. However, certain variants may lead to serious nucleotide changes, causing aberrant enzymatic activity and abnormal biochemical reactions, and in some cases, a shift towards malignancy and carcinogenesis (Day et al., 2003; Huang et al., 2021).

In contrast to variants, which are relatively common in the human population, mutations represent abnormal, permanent changes that occur due to lesions at the DNA structure level in specific individuals, with a prevalence of less than 1% in the population.

**FIGURE 1:**
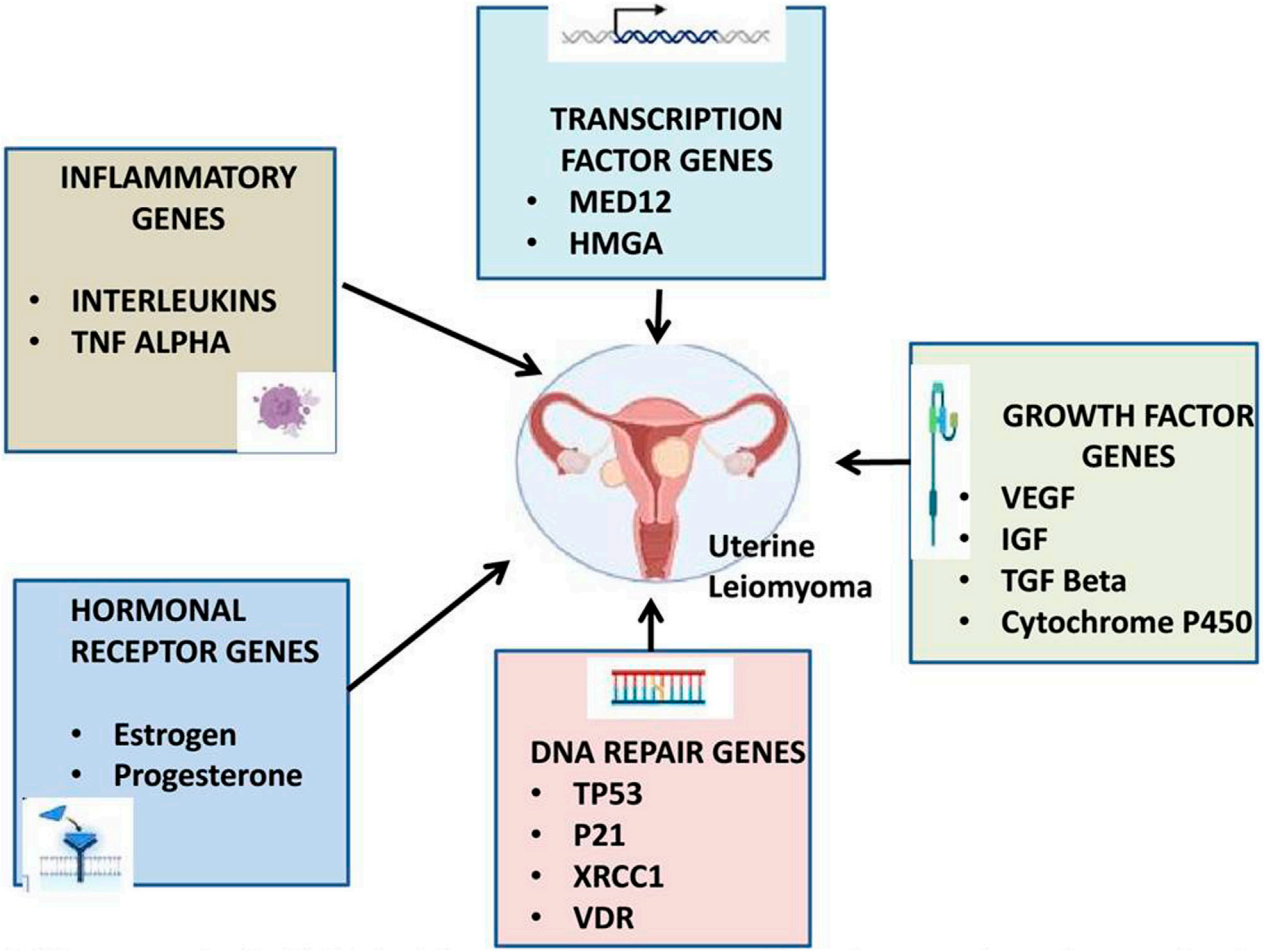
Different Genes involved in uterine Leiomyoma Cause. The present illustration summarizes various genetic and epigenetic factors are involved behind uterine leiomyoma susceptibility..

Over the years, the scientific community has been fascinated by the investigation of the connection between genetic variation and cancer risk, mainly due to the potential to develop personalized treatments using more targeted therapies based on individual genomic profiles. Numerous studies in the literature have explored the link between variants in different genes and uterine leiomyoma, but interpreting the findings can be challenging due to the inconsistency of results. Figure 1 illustrates the different Genes behind Uterine Leiomyoma cause.

### Gene variants involved in DNA repair, apoptosis and genomic stability

#### Variants of TP53 and p21 gene

Uterine leiomyoma originates from the uncontrolled growth of a single myometrial cell, indicating its monoclonal nature. Consequently, the disruption in the regulation of the cell cycle might play a crucial role in the initiation and progression of this tumor (Day et al., 2003). The role of p21 gene SNPs in UL was investigated by Salimi et al. who evaluated Iranian women carrying the CA genotype exhibited a 1.8-fold elevated risk of UL. There was a notable increase (28%) in the occurrence of the CA genotype of the p21 C^9^8A variant when contrasted with the control group (18%). However, no association was found between UL and rs1059234 (Salimi et al., 2016). On other hand, Hseieh et al. reported alleles related to p21 codon is connected with the presence of leiomyomas.

Aberrant p53 expression has been identified as a significant factor contributing to the development of various types of tumors. Hseieh et al. revealed that among the identical sequence variations situated within the promoter region of the p53 gene., the SNPs at positions −216C and −103G have been linked to the development of leiomyomas. Moreover, Hseieh et al. also found that the gene variant at p53 codon 72 does not exhibit any connection with the susceptibility to leiomyomas in Taiwanese women (Hsieh et al., 2004). Dominik Denschlag et al. also reported that the presence of the p53 variant at codon 72 is indicative of the likelihood of susceptibility to leiomyoma within a Caucasian population, potentially playing a role in the development of uterine leiomyoma (Denschlag et al., 2005). The both above mentioned studies are different on the basis of population type and number of samples collected which we think may be the main reason behind such contradictory outcome. However, one of the study revealed a notable elevation in estrogen levels, ER-α receptor expression, and corresponding mRNA in leiomyoma cells compared to normal myometrium. Estrogens play a crucial role in promoting the growth of fibroid by enhancing the production of cytokines and growth factors, while simultaneously inhibiting apoptosis through the suppression of the p53 gene (Thompson et al., 1990). Thus, these all studies indicate that variants of p53 genes play pivotal role in promoting the growth of uterine leiomyoma.

#### Variants of XRCC1 gene

The X-ray repair cross-complementing group 1 (XRCC1) proteins play a pivotal role in maintaining genome stability through their involvement in base excision repair, a crucial DNA repair pathway (Liang et al., 2015). One study reported that in Iraqi Arabian women, a noteworthy and positive correlation was observed between the XRCC1 gene variant (rs25487) and the development of uterine leiomyoma. Specifically, individuals with the recessive GG genotype were potentially seven times more prone to uterine leiomyoma development compared to those with the combined (AG + AA) genotypes. The odds ratio stood at 7.59 with a 95% confidence interval of 3.77–15.25 (Tajuldeen et al., 2019). On other hand, Yang et al. investigated the presence of the XRCC1 Arg280His variant was linked to an elevated risk of uterine leiomyoma within a Chinese population (Yang et al., 2010). But according to Hsieh et al. no correlation was found between XRCC1 codon 399 variant and UL in Taiwan Chinese women. However, Jeon et al. found that among Korean women, the presence of the XRCC1 399Gln variant is linked to a heightened risk of uterine leiomyoma (Jeon et al., 2005).

This portion of the study specifically examines the XRCC1 gene, whose product plays a crucial role in DNA repair. Despite not possessing intrinsic enzymatic activity, XRCC1 actively engages with enzymatic components involved in various stages of DNA strand break repair, such as PARP-1, AP endonuclease-1, polynucleotide kinase, DNA polymerase-β, and DNA ligase IIIα. Consequently, polymorphisms in XRCC1 that lead to amino acid substitutions have the potential to disrupt the interaction between XRCC1 and other enzymatic proteins, thereby affecting the process of DNA strand break repair (Holick, 2003).

#### Variants of the VDR gene

In contemporary times, there is growing recognition that vitamin D deficiency constitutes a significant risk factor in the progression of UL. On average, the serum levels of 25-hydroxyvitamin D (25(OH)D) are noticeably lower in women who have UL compared to those without UL (Wise et al., 2014). Commencing in 2014, a team of researchers initiated an investigation into the association between SNP gene variants and the occurrence of UL. Initial examinations indicated that SNPs linked to vitamin D metabolism and skin color are connected to the presence of UL in black women. Notably, among the scrutinized SNPs, rs12800438 near the DHCR7 gene and rs6058017 in the ASIP gene are implicated in vitamin D synthesis in the skin. Furthermore, an association has been established between UL and the variants rs739837 and rs886441 in the nuclear hormone receptor for vitamin D (Wise et al., 2014). Shahbazi et al.'s research lends support to the idea that VDR rs2228570 variant is related to UL—specifically, a correlation between the VDR TT genotype and an elevated risk of UL occurrence (Shahbazi, 2016). Further, Yilmaz et al. demonstrated that the presence of the rs2228570 CC genotype might act as a risk-reducing factor, while the T allele could potentially contribute to the risk of UL, aligning with Shahbazi’s findings (Yılmaz et al., 2018). However, one of the findings indicated that there is no observed correlation between the VDR variants rs731236, rs1544410, and rs2228570, and the incidence of uterine leiomyoma in Caucasian women (Ciebiera et al., 2019). Vitamin D plays a crucial role in the development of the extracellular matrix (ECM) growth. Leiomyoma cells exhibit a markedly higher level of production for the primary components of ECM, including collagens, fibronectin, and proteoglycans, compared to normal myometrium cells. Nevertheless, additional investigations are required to evaluate the actual significance of VDR variants in the etiopathogenesis of uterine leiomyomas.

### Variants of transcription factor genes

#### Variants of the MED12 gene

Genetic mutations in MED12, a constituent gene of the mediator complex, have been identified primarily within exon 2. These mutations are recognized as the driving force behind the development of uterine leiomyomas across diverse ethnic groups, including Caucasian, African American, and Asian women (Halder et al., 2015).

In a recent study by Markowski and colleagues, an examination was conducted on the molecular characteristics of MED12 deletions across 70 smooth muscle tumors. These deletions encompassed a genomic segment of 72 base pairs, commencing within intron 1 and concluding at position c.166 of exon 2. The researchers postulated that these deletions arise as a consequence of non-B DNA structures located within the hotspot region of deletion. Similar to many other oncogenes, the majority of MED12 mutations are consistently found at a specific amino acid location, codon 44. This recurrence of mutations at this particular position implies that glycine at codon 44 serves as a hotspot for mutations in leiomyomas (Tomlinson et al., 2002). Moreover, the fact that this location falls within evolutionarily conserved nucleotide sequences further suggests that it might be a potential causative variant, as indicated by various studies (Roy et al., 2006; Mehine et al., 2013; Ajabnoor et al., 2018). Around 85% of sporadic uterine fibroids (UFs) have exhibited an increased occurrence of somatic mutations in the MED12 gene, which encodes the transcriptional mediator complex subunit 12. These mutations commonly include the c.131G>A missense variant. Additionally, these fibroids tend to exhibit distinct characteristics such as notable chromosomal loss and rearrangement (Mehine et al., 2013).

Mutations in the MED12 gene as discussed above have been demonstrated to impair its activation of cyclin C-dependent CDK8 and its stimulatory activity on CDK19. Additionally, these mutations have been linked to the abnormal activation of numerous genes that regulate crucial pathways in leiomyoma pathogenesis. These pathways include Wnt/β-catenin signaling, hedgehog signaling, sex steroid receptor signaling, and the transforming growth factor (TGF)-β receptor signaling pathways suggest that genetic variations in these genes could lead to genesis of leiomyoma through modulating various signaling pathways.

### Variants of growth factor genes

#### Variants of vascular endothelial growth factor

VEGF, a peptide with angiogenic properties, holds a significant role in promoting the growth of various tumors. Among the seven distinct variations of VEGF (VEGF-A, -B, -C, -D, -E, -F, and placental growth factor), VEGF-A and VEGF-B are primarily associated with angiogenesis. However, there exists only a single study that has explored the potential link between the VEGF-460 variant located in the 5′-untranslated region of the VEGF gene and uterine leiomyomas (UL) (Andaloussi EI et al., 2020). Farshid Keshavarzi et al. indicated that the VEGF-2578C/A (rs699947) variant showed no correlation with uterine leiomyomas (UL). On the other hand, a notable association was observed between the insertion/deletion (I/D) variant at position −2,549 in the promoter region of the VEGF gene and uterine leiomyomas (Keshavarzi et al., 2017). Hsieh et al. investigated that the individuals with the homozygous T genotype and the T allele of the VEGF gene −460 variant exhibit an increased susceptibility to the development of leiomyomas (Hsieh et al., 2008).

Vascular endothelial growth factor (VEGF) stands out as a crucial angiogenic growth factor, playing a significant role in the regulation of angiogenesis and the mediation of sex steroid-induced cell growth and differentiation. The activities mediated by VEGF appear to play a role in the pathogenesis of leiomyoma. Genetic variations, encompassing polymorphisms, in VEGF may also be linked to the intricate pathogenic mechanisms underlying leiomyomas.

#### Variants of IGF and TGF beta genes

Transforming growth factor ß (TGFß) is widely acknowledged as a pivotal factor in the aberrant growth of fibrotic tissue, particularly in the context of leiomyomata (Biernacka et al., 2011). Veronica M et al. found that the TGF β1 -509 C/T variant holds the potential to serve as a marker for susceptibility in the pathogenesis of uterine leiomyoma (Veronica et al., 2016).

IGF2 is a recognized stimulator of cell proliferation and has been newly identified as a marker specific to fibroids. Vaidya et al. investigated that the IGF2 ApaI variant within the gene was examined. The presence of the ‘G' allele was notably linked to uterine leiomyomas (odds ratio = 3.56; 95% confidence interval (2.454–5.172), *p* < 0.0001) (Vaidya et al., 2017).

Mitogenic growth factors, including transforming growth factor, basic fibroblast growth factor, epidermal growth factor, and insulin-like growth factor-I, are heightened in fibroids and could act as effectors in response to estrogen and progesterone stimulation. Mutations associated with these growth factors provide insights into the etiology and pathogenesis of uterine leiomyomas. Still, it is essential for forthcoming research to find the variants of IGF and TGF beta genes as targets for uterine leiomyoma treatment.

### Variants of metabolic genes

#### Variants of fumarate hydratase gene

The Fumarate Hydratase (FH) gene is responsible for encoding fumarate hydratase, a crucial enzyme within the Krebs cycle. Operating as a homotetramer, it facilitates the conversion of fumarate into malate through hydration. When mutations occur in the FH gene, they give rise to a rare autosomal dominant inherited metabolic disorder, leading to the formation of uterine leiomyomas (Gregová et al., 2020). Ping Li et al. reported that the c.557G>A (p.S186N) mutation in the FH gene was detected within a Chinese family displaying the phenotype of uterine leiomyomas (Li et al., 2022). Similarly, Jorge R. Toro et al. reported that a shared mutation, R190H, was found among eleven distinct unrelated families of North America (Toro et al., 2003). Zichen Zhao et al. also investigated through their whole exome sequencing an unreported missense mutation in the FH gene (c.1214A>G, p. Leu405Ser) which was detected in both the patients and their father (Zhao et al., 2020). The study summarized that alteration in FH gene may lead to leiomyoma cause. Still further investigation needed to find the etiology behind the leiomyoma growth.

#### Variants of cytochrome P450 gene

The group of enzymes known as Cytochrome P 450 plays a role in both the metabolic activation of carcinogens and the biosynthesis of steroid hormones (Sowers et al., 2006). Yi Ye et al. reported through their findings that the presence of the CYP1A1 Ile462Val genotype was linked to a higher susceptibility to uterine leiomyomas among Chinese women (Ye et al., 2008). Likewise, D Herr et al. investigated the correlation between the presence of uterine leiomyoma and two single nucleotide variants (SNPs) within the genes CYP2A13 and CYP1A1 in a Caucasian population. Among women diagnosed with uterine leiomyoma and the control group, the allele frequencies of CYP2A13 were 3.6% and 1.2%, respectively, for the T allele, while for the C allele, they were 96.4% and 98.8%, respectively. The variant 3375C > T results in an amino acid alteration from Arginine (Arg) to Cysteine (Cys) at position 257 in the anticipated amino acid sequence (Herr et al., 2006). Some researchers demonstrated a notable link between the CYP1B1 Leucine432Valine SNP and the presence of uterine leiomyoma among Black women (Bideau and Alleyne, 2016). Different studies indicated the association of CYP1A1 variants behind Leiomyoma cause. Still more research needed to find variants in regards to UL susceptibility.

#### Variants of hormonal receptor genes

Based on reliable and comprehensive data, the expansion of fibroids is primarily contingent on steroid hormones. Elevated levels of estrogen and progesterone are considered among the foremost influential factors triggering the genesis and progression of uterine leiomyoma.

The interplay between progesterone and estrogen, their respective receptors, and other paracrine signals is integral to the etiopathogenesis of uterine leiomyomas (uLM). Progesterone has the capacity to suppress estrogen receptors. Conversely, disruptions in estrogen levels can impact the functionality of both estrogen and progesterone-associated genes and pathways.

Estradiol, the primary form of estrogen, can initiate the production of specific growth factors, particularly platelet-derived growth factor (PDGF), through the MAPK-PKC pathway. This cascade ultimately results in heightened proliferation or immortalization. Furthermore, estradiol is capable of activating the Wnt/β-Catenin pathway through estrogen receptor alpha (ERα), promoting cellular proliferation. Notably, similar to progesterone, strategies for inhibiting estrogen activity, particularly through the use of aromatase inhibitors, have been proposed. These findings underscore the pivotal role of both progesterone and estrogen in the pathogenesis of uterine leiomyomas.

#### Variants of estrogen receptor gene

Two primary intracellular estrogen receptors, namely, ER-α and ER-β, have been recognized. These receptors are encoded by separate genes, ESR1 and ESR2, and function as hormone nuclear receptor proteins. They act as ligand-inducible transcription factors, each exhibiting distinct tissue expression patterns, post-translational modifications, and cellular localization in both normal and disease conditions.

Hsieh et al. noted that the presence of the rs2234693C allele and the associated genotypes were linked to an increased susceptibility to both endometriosis and uterine fibroids (Hsieh et al., 2005). Fischer et al. investigated that the presence of three SNPs in the promoter region of the ESR2 gene (rs2987983, rs3020450, and rs3020449) is not linked to the development of uterine fibroids (Fischer et al., 2009). Xian-Dun Zhai et al. conducted an examination of two prevalent ESR2 variants, rs1256049 (G1082A) and rs928554 (Cx + 56 A → G), to determine their potential connection with the risk of uterine leiomyoma. This investigation involved the use of polymerase chain reaction–restriction fragment length variant and DNA sequencing methods (Zhai et al., 2009). Fabiola E. Villanova et al. identified the occurrence rate of two single nucleotide variants (SNPs): one situated within intron 1 (rs9322331) and another in exon 1 (rs17847075) of the estrogen receptor α (ESR1) gene in uterine leiomyoma. One of the study revealed the presence of TC and CC genotypes of the ESR1 rs2234693 variant could potentially lower the susceptibility to uterine fibroids (UFs), particularly among premenopausal women (Villanova et al., 2006). Dominik Denschlag et al. investigated the presence of the ESR1 IVS1-397T/C (PvuII) SNPs does not exhibit any correlation with the vulnerability to uterine leiomyoma within a Caucasian population (Tajuldeen et al., 2019). Though, this result is contradictory which cannot be included as final decision as it has small sample size but as part of the study. However, more findings needed to find the actual cause behind leiomyoma.

#### Variants of progesterone receptor gene

Stefan P. Renner et al. found no noteworthy correlation was discovered between the +331G/A or the V660L SNPs and the likelihood of myoma development (Renner et al., 2008). This study is although contradictory in specific population and sample size but still further investigation is needed to be performed in other populations to reach to any conclusion. One of the study investigates the potential influence of estrogen and progesterone plasma levels in relation to the gene variants of ERβ (−13950T/C) and PGR (+331G/A) receptor genes on the risk factors (Veronica et al., 2016). There is a scarcity of available information in the existing literature concerning genetic variations within the Progesterone receptor genes. Therefore, it is essential for forthcoming research to address this subject, particularly considering the potential heightened risk of malignancy associated with single nucleotide variants (SNPs) present in this gene.

#### Variants of genes involved in inflammation

The connection between inflammation and cancer has been extensively explored by multiple researchers, establishing its connection to cancer incidence, metastasis, and resistance to treatment. Consequently, directing focus towards genes implicated in the inflammatory process could hold significant potential for cancer control.

Cytokines, particularly certain interleukins, have a pivotal function in orchestrating interactions between immune and non-immune cells within the tumor microenvironment. This interaction significantly impacts cancer’s evolution and advancement.

#### Variants of interleukin genes

Interleukins and other cytokines have demonstrated their involvement in influencing susceptibility to a range of gynecological neoplasms (Paltridge et al., 2013). A notable disparity was observed in the allele frequencies of the IL-1b-511 C < T variant by Pietrowski et al. (Pietrowski et al., 2009). Moreover, one of the studies revealed the IL-12Rβ1 codon 378G homozygote and the presence of the G allele are associated with an increased susceptibility to leiomyoma. However, there is no observed correlation between leiomyoma development and gene variants in IL-1β-511 promoter, IL-1β exon 5, IL-1Ra, IL-2 114, IL-4 −590 intron 3, IL-8 3′-UTR 2767, and IL-18 105. Mortezaee et al. investigated the association of IL-alpha (−511C>T) variant with uterine leiomyoma in Iranian women (Shariati et al., 2015). The rs20541 variant (IL13) potentially plays a role in increasing susceptibility to the development of uterine leiomyomas (ULM), while the rs1801275 variant (IL4R) could predispose individuals to the occurrence of multiple ULM as indicated by one of the findings (Krsteski et al., 2017). This contradiction in the two findings is based on the different population and sample sizes taken in their particular study. The conclusion cannot be made on these small and limited population, further findings needed in other population to reach the final conclusion. In the investigation conducted by Sosna et al., the identical rs2070874 (IL4) variant was examined, revealing an observed link with the susceptibility to uterine leiomyomas (Sosna et al., 2010). SNPs related to interleukins could impact the production of interleukins, influencing the progression of the condition and playing a role in both disease resistance and susceptibility.

In conclusion, the presence of a single nucleotide variant (SNP) within the IL-genes could potentially play a significant role in the development of uterine leiomyoma.

#### Variants of tumor necrosis factor gene

Tumor necrosis factor-alpha (TNF-α), a cytokine known for inducing inflammation, holds a significant role in various immune system disorders and is implicated in both tumor development and progression (Jang et al., 2021). Medikare et al. reported the presence of the “TC” genotype and the “C” allele in rs1799964 (−1031T/C) is linked to an elevated susceptibility to leiomyomas. The “C” allele of −1031T/C contributes to heightened TNF-α expression, fostering smooth cell proliferation and tumor advancement. As a result, this allele could serve as a meaningful molecular marker for identifying and understanding uterine leiomyomas (UL). In addition to IL-4, we identified distinct genotype distributions in the TNFA gene −308 A/G. Notably, the frequency of the AA genotype was more prevalent in the group of younger patients (≤35 years), exhibiting a statistically significant difference (*p* = 0.02) (Medikare et al., 2015). However, our understanding of this topic is still limited, and further research is needed to explore TNF-α dependent pathways in the pathophysiology of uterine fibroids.

#### Variants of genes involved in folate metabolism

Folate metabolism plays a role in multiple functions like synthesis, methylation, and DNA repair. Key genes such as methylenetetrahydrofolate reductase (MTHFR), methionine synthase (MTR), reduced folate carrier 1 (RFC1), and cystathionine β-synthase (CßS) are crucial in governing this metabolic pathway. Modifications in these genes may result in genetic instability, potentially elevating the likelihood of carcinogenesis (Duthie, 2011). Jiahui Shen et al. reported through their findings an association between the MTHFR C677T variant and the occurrence of uterine leiomyoma for the first instance (Shen et al., 2021). This suggests a potential link that could elevate the likelihood of uterine fibroid development in women during their gestational period.

In prior investigations, the overall frequency of the 677T allele was established at 45.2% in China, a figure surpassing that of numerous other countries (Yang et al., 2013). In another study, a higher prevalence of heterozygous mutations (C/T) (1011/2411) compared to homozygous mutations (T/T) (328/2411) was observed. The MTHFR gene, a pivotal enzyme in folate metabolism, when mutated, could potentially diminish the activity of folate-metabolizing enzymes, elevate plasma homocysteine (Hcy) levels, induce oxidative stress, and foster the accumulation of extracellular matrix in fibroids. Consequently, the heterozygous mutations (C/T) may manifest as the most noteworthy phenotype (Wang et al., 2016). Thus, variants of MTHFR gene may lead to variation and elevate the growth of leiomyoma in utreus.

The discussed factors and its associated variants as summarized in Table 1 indicates how these changes play a pivotal role behind uterine Leiomyoma cause. Although much more investigation is needed regarding variants to associate with the pathophysiology of uterine Leiomyoma.

**TABLE 1:** Summary of variants of different genes behind the cause of uterine Leiomyoma.

Variants	Genes involved	References
C98A	P21	Salimi et al. (2016)
rs25487	XRCC1	Tajuldeen et al. (2019)
Arg280His	XRCC1	Yang et al. (2010)
399 Gln	XRCC1	Jeon et al. (2005)
rs739837 and rs886441	XRCC1	Jeon et al. (2005)
rs2228570	VDR	Shahbazi (2016), Yilmaz et al. (2018)
c.131G>A	MED12	Mittal et al. (2015), Andaloussi Ei et al. (2020) Pandey et al. (2024)
2578C/A (rs699947)	VEGF	Keshavarzi et al. (2017)
T allele of the VEGF gene −460 variant	VEGF	Hsieh et al. (2008)
−509 C/T	TGF β1	Veronica et al. (2016)
c.557G>A	FH	Li et al. (2022)
c.1214A>G, p.Leu405Ser	FH	Zhao et al. (2020)
R190H	FH	Toro et al. (2003)
3375C > T	CYP2A13	Herr et al. (2006)
rs2234693	ER	Hsieh et al. (2005)
rs1256049 (G1082A) and rs928554 (Cx + 56 A → G)	ER	Zhai et al. (2009)
rs9322331,rs17847075	ER	Villanova et al. (2006)
511 C < T	IL	Pietrowski et al. (2009)
rs2070874	IL4	Sosna et al. (2010)
rs1799964	TNF	Medikare et al. (2015)
C677T	MTHFR	Shen et al. (2021)

#### Future perspective

Uterine leiomyoma, much like other forms of tumors, is characterized by a complex interplay of genetic alterations. Genetic investigation of cancer has held significant importance for the scientific community over recent years. The outcomes of both existing and forthcoming research could provide insights into the underlying biological and pathological mechanisms driving this disease. This knowledge may facilitate the identification of therapeutic targets and biomarkers, potentially contributing to strategies for preventing uterine leiomyoma or tailoring individualized approaches to diagnosis and treatment, thus enhancing prognosis prospects.

This review underscores the examination of numerous single nucleotide variants (SNPs) across different genes concerning their connections to susceptibility, morpho-pathological characteristics, and prognostic implications in uterine leiomyoma. However, several questions remain unanswered regarding the precise biological mechanisms underlying the effects of genetic variants. Despite this, the abundance of statistically significant outcomes across the literature strongly supports their involvement in leiomyoma. Additionally, it is evident that numerous other SNPs could influence not only the genetic predisposition to uterine fibroid but also the clinical and histopathological characteristics of the disease.

Future research should take specific directions to further advance our understanding in this area. Larger cohorts should be incorporated, with a heightened emphasis on considering factors like ethnicity and environmental influences. Improved study designs are crucial to comprehensively grasp the impact of these genetic variants on uterine leiomyoma. Another critical avenue of investigation is to intricately define the cancer phenotype, including tumor histological subtypes, behavior, aggressive clinical and histopathological traits, and their associations with specific genetic variants.

The exploration of single nucleotide polymorphisms (SNPs) has the potential to pave the way for the development of novel therapies and prognostic biomarkers. For instance, identifying specific SNPs associated with drug metabolism can aid in personalized medicine, allowing for tailored drug treatments based on an individual’s genetic makeup. Additionally, understanding SNPs related to disease susceptibility or progression can contribute to the development of targeted therapies aimed at addressing specific genetic factors underlying certain conditions. This approach aligns with the broader field of precision medicine, where interventions are customized to an individual’s genetic profile for more effective and personalized medical care.

## Conclusion

Over the past few decades, significant strides have been taken in the realm of genetic analysis within uterine leiomyoma research. The insights derived from genetic studies hold a crucial role in unraveling the underlying mechanisms of uterine fibroid pathogenesis, and potentially contribute to the identification of novel diagnostic and prognostic biomarkers. Additionally, the examination of genotypes and the presence of single nucleotide variants (SNPs) across various genes could empower clinicians to customize personalized approaches to management, as well as provide valuable insights for individuals at risk of developing uterine leiomyoma.

Nonetheless, the current body of literature on genetic variants necessitates further enrichment through continued research efforts. Ideally, these endeavors should involve larger cohorts, while also taking into account the diverse ethnic backgrounds and environmental factors that influence leiomyoma development. This comprehensive approach is pivotal for a more profound comprehension of the oncogenic mechanisms at play in uterine leiomyoma.
